# Identification of a novel non-coding deletion in Allan-Herndon-Dudley syndrome by long-read HiFi genome sequencing

**DOI:** 10.1186/s12920-024-02058-4

**Published:** 2025-03-03

**Authors:** Jihoon G. Yoon, Seungbok Lee, Soojin Park, Se Song Jang, Jaeso Cho, Man Jin Kim, Soo Yeon Kim, Woo Joong Kim, Jin Sook Lee, Jong-Hee Chae

**Affiliations:** 1 Department of Genomic Medicine, Seoul National University Hospital, Seoul, Republic of Korea; 2 Department of Laboratory Medicine, Gangnam Severance Hospital, Yonsei University College of Medicine, Seoul, Republic of Korea; 3 Department of Pediatrics, Seoul National University College of Medicine, Seoul, Republic of Korea; 4 Department of Pediatrics, Seoul National University Bundang Hospital, Seongnam-si, Gyeonggi-do Republic of Korea; 5 Department of Laboratory Medicine, Seoul National University Hospital, Seoul, Republic of Korea

**Keywords:** Allan-Herndon-Dudley syndrome, MCT8, *SLC16A2*, Long-read sequencing, Non-coding deletion, Transcription factor

## Abstract

**Background:**

Allan-Herndon-Dudley syndrome (AHDS) is an X-linked disorder caused by pathogenic variants in the *SLC16A2* gene. Although most reported variants are found in protein-coding regions or adjacent junctions, structural variations (SVs) within non-coding regions have not been previously reported.

**Methods:**

We investigated two male siblings with severe neurodevelopmental disorders and spasticity, who had remained undiagnosed for over a decade and were negative from exome sequencing, utilizing long-read HiFi genome sequencing. We conducted a comprehensive analysis including short-tandem repeats (STRs) and SVs to identify the genetic cause in this familial case.

**Results:**

While coding variant and STR analyses yielded negative results, SV analysis revealed a novel hemizygous deletion in intron 1 of the *SLC16A2* gene (chrX:74,460,691 − 74,463,566; 2,876 bp), inherited from their carrier mother and shared by the siblings. Determination of the breakpoints indicates that the deletion probably resulted from Alu/Alu-mediated rearrangements between homologous AluY pairs. The deleted region is predicted to include multiple transcription factor binding sites, such as Stat2, Zic1, Zic2, and FOXD3, which are crucial for the neurodevelopmental process, as well as a regulatory element including an eQTL (rs1263181) that is implicated in the tissue-specific regulation of *SLC16A2* expression, notably in skeletal muscle and thyroid tissues.

**Conclusions:**

This report, to our knowledge, is the first to describe a non-coding deletion associated with AHDS, demonstrating the potential utility of long-read sequencing for undiagnosed patients. Although interpreting variants in non-coding regions remains challenging, our study highlights this region as a high priority for future investigation and functional studies.

**Supplementary Information:**

The online version contains supplementary material available at 10.1186/s12920-024-02058-4.

## Background

Allan-Herndon-Dudley syndrome (AHDS; OMIM #300523), also known as monocarboxylate transporter 8 (*MCT8*) deficiency, is an X-linked disorder caused by pathogenic variants in the solute carrier family 16 member 2 (*SLC16A2*) gene [1]. This transmembrane transporter is crucial for transporting the active form of thyroid hormone, triiodothyronine (T3), which is critical for neurodevelopmental processes. The *MCT8* deficiency can lead to severe psychomotor deficits that include hypotonia, muscle hypoplasia, spastic paraplegia, intellectual disability, movement disorders, and microcephaly often with abnormal thyroid function test (TFT) results such as high free T3, low free T4, and normal or slightly elevated TSH concentrations [2, 3]. While the vast majority of AHDS patients are male, a few cases have been reported in females due to defects in X-inactivation processes [4–6]. Causative variants of AHDS have typically been identified in protein-coding regions or adjacent junctions. Furthermore, structural variations (SVs), such as deletions involving *SLC16A2* coding regions, have also been described [7–10]. However, non-coding deletions in AHDS have not been documented to date.

Recently, advances in long-read sequencing technologies, such as PacBio SMRT sequencing or Oxford Nanopore sequencing, have enabled the detection of the full spectrum of human genetic variations, leading to the discovery of novel disease mechanisms [11]. Particularly, long-reads with read lengths generally exceeding 10 kb have advantages over short-reads for the detection of complex SVs and short-tandem repeats (STRs), and for variant phasing [12–14]. Some studies have demonstrated their clinical utility as a diagnostic approach in patients undiagnosed by short-read sequencing, especially in Mendelian disorders [15–17]. Therefore, we adopted long-read HiFi genome sequencing for two male siblings with severe neurodevelopmental problems, who were previously negative by exome sequencing. As a result, we identified a novel association between a *SLC16A2* non-coding deletion and this familial case.

## Materials and methods

### Subjects

The two affected siblings visited our outpatient clinics at the Rare Disease Center of Seoul National University Children’s Hospital. They underwent chromosomal microarray and exome sequencing in a quartet for genetic diagnosis. However, previous genetic tests had yielded negative results including copy-number variation (CNV) and STR analysis [18]. Informed consent was appropriately obtained from their parents, and this study was approved by the Institutional Review Board of Seoul National University Hospital (IRB No.1406-081-588).

### Long-read HiFi genome sequencing and analysis

Whole blood was obtained from the probands and their family members. Long-read genome sequencing was performed on the genomic DNA samples obtained from the two siblings. The sequencing protocol followed the previously described method [17]. Briefly, the genomic DNA was extracted and fragmented to achieve a target size of 14 kb. SMRT bell libraries were prepared using the SMRTbell Express Template Prep Kit 2.0 (100-938-900, Pacific Biosciences, USA) following the manufacturer’s protocol. The libraries were sequenced on the Sequel IIe System, aiming for an average coverage of 10x, utilizing the Sequel II Binding Kit 2.2 (102-089-000) and Sequel II Sequencing Kit 2.0 (101-820-200; Pacific Biosciences, USA).

For the analysis of the sequenced reads, we utilized the PacBio Human WGS workflow (https://github.com/PacificBiosciences/pb-human-wgs-workflow-snakemake) developed in snakemake. The HiFi reads were aligned to the human reference genome hg38 using ‘pbmm2’, and variant detection and joint genotyping were conducted using Deepvariant and GLnexus [19]. SVs and STRs were identified using ‘svpack’ and ‘tandem-genotypes’, respectively [20]. We utilized AnnotSV [21] and the gnomAD SV v4.0 database [22] to prioritize the detected SVs.

### Multiplexed PCR, Sanger sequencing, and RT-PCR

The identified *SLC16A2* intronic deletion was confirmed using a multiplexed PCR method, which allowed for the simultaneous amplification of both the wild-type (WT) and deleted alleles. The fragment size of WT and deleted alleles were designed to be 373 bp and 852 bp, respectively. The PCR was carried out using the AccuPower^®^ Taq PCR PreMix & Master Mix (Bioneer, Korea) following the manufacturer’s protocol, with genomic DNA samples obtained from the two patients and their parents. The sequence information of the primers is as follows: F1 (5’-TGTACCCAGGGTCTAGCCTTG-3’), R1 (5’-CAATGTTTAGAAACCTGCTGAATG-3’), R2 (5’-ACATTTCCCAA‌‌‌‌G‌TCAGTTTCTGCT-3’). The amplified PCR products were visualized by electrophoresis on a 1.5% agarose gel. The breakpoint was identified through Sanger sequencing of the PCR product containing the deleted allele. The impact of the deletion on aberrant splicing between exons 1 and 2 was assessed through RT-PCR from blood-derived mRNA using the primers as follows: F (5’-GAAACAAGTACCAGCCACAAAG-3’), R (5’-GAGAATCCCGTAGGTGAAGTAG-3’).

### Characterization of the *SLC16A2* intronic deletion

Genomic information concerning the identified *SLC16A2* intronic deletion was obtained using the UCSC Genome Browser (https://genome.ucsc.edu/). Transcription factor (TF) binding sites were predicted by the JASPAR database [23]. Tissue-specific gene expression data for the *SLC16A2* gene were analyzed using the Genotype-Tissue Expression (GTEx) database (https://www.gtexportal.org/) [24]. The expression quantitative trait loci (eQTL; rs1263181) located within the intronic deletion was explored for the 49 available tissues. Annotation information for regulatory features was obtained from RegulomeDB (https://regulomedb.org/) [25].

## Results

### Clinical findings

**Fig. 1:**
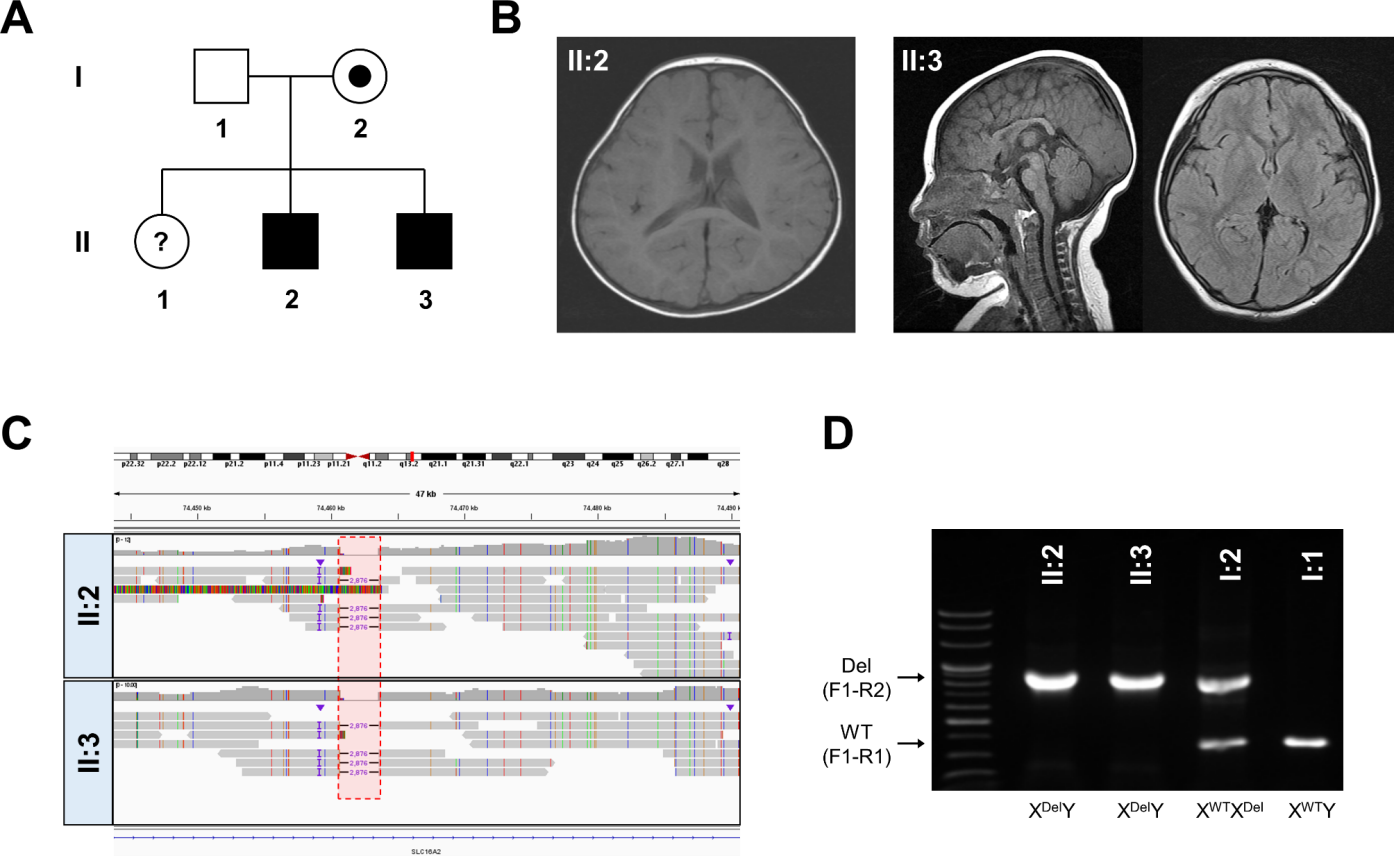
Clinical and genomic characterization of a familial case of Allan-Herndon-Dudley syndrome. **A**. Pedigree of the family with two affected male patients (II:2, II:3) presented with global developmental delay, early onset seizures, intellectual disability, and post-natal microcephaly. **B**. Brain imaging findings of affected individuals II:2 (left, axial view) and II:3 (middle, sagittal view; right, axial view) showing normal structural brain anatomy with suggestive findings of hypomyelination in patient II:3. **C**. Integrative Genomic Viewer findings of long-read HiFi genome sequencing. It reveals a hemizygous intronic deletion in the *SLC16A2* gene shared by the two affected siblings. The deleted region is highlighted in red. **D**. The genotyping result of the *SLC16A2* intronic deletion using a multiplexed PCR method in the family members. The fragment size of wild-type (WT) and the deletion alleles were 373 bp (F1-R1) and 852 bp (F1-R2), respectively. The genotype results confirmed the carrier status in the asymptomatic mother

The family presented with one unaffected female and two affected male siblings displaying similar symptoms, strongly suggestive of a genetic disorder with a potential autosomal or X-linked recessive mode of inheritance (Fig. 1A). The first child (II:1), a female, showed normal development without any medical complications. The second child (II:2) was born prematurely at 36 weeks of gestation and had a birth weight of 1.64 kg. Shortly after birth, he experienced severe respiratory distress, infantile spasms, and seizures. He also had severe global developmental delay and spasticity. However, brain magnetic resonance imaging (MRI) did not reveal any significant abnormalities (Fig. 1B). A TFT performed at 14 months of age showed abnormal results, including elevated serum T3 levels (214 ng/dl, reference range: 87–184) and increased TSH levels (4.83 uIU/ml, reference range: 0.4–4.1), while free T4 levels remained within the normal range (1.11 ng/dl, reference range: 0.7–1.8). He also exhibited post-natal microcephaly (Z-score − 4.20) and severe growth delay, with his height and body weight at six years of age measuring 97.6 cm (Z-score − 1.92) and 15 kg (Z-score − 2.59), respectively.

The third child (II:3), who is the younger brother of II:2, was born at 40 weeks of gestation, with a birth weight of 3.20 kg. Unlike his brother, he did not experience any significant perinatal problems. However, he also showed severe global developmental delay, post-natal microcephaly (Z-score − 4.64), and severe failure to thrive. Similar to his brother, he also had a history of infantile spasms and seizures. His TFT results assessed at eight months of age fell within the normal range (total T3: 155 ng/dl, TSH: 1.62 uIU/ml, free T4: 0.95 ng/dl). Brain MRI findings revealed suggestive findings of thinning of the corpus callosum and hypomyelination, although no remarkable brain anomalies were detected (Fig. 1B). At 14 years old, his height and body weight remained significantly below the normal range, measuring 76.6 cm (Z-score − 8.19) and 10 kg (Z-score − 16.82), respectively. Currently, both patients are 21 and 17 years old, with severe intellectual disability and spasticity and controlled seizures under anti-epileptic drugs without regression.

### Genomic analysis

These two siblings had remained undiagnosed for over a decade, even after undergoing several genetic tests, including karyotyping, CMA, and exome sequencing. To explore other potential genomic variations that are not sufficiently covered by exomes, such as SVs or STRs, we employed long-read HiFi sequencing. Analysis of SNVs or known 52 pathogenic STR loci (Figure S1) did not yield any diagnostic clues, similar to the results of exome sequencing. There were no OMIM genes with one allele having a single pathogenic or likely pathogenic SNV and the other allele having an SV in compound heterozygotes. Considering the suspected inheritance mode of autosomal or X-linked recessive disorders, we focused on homozygous or hemizygous alleles shared by both siblings, specifically targeting SVs larger than 50 bps. Employing stringent criteria, we sought SVs within OMIM genes, requiring a minimum depth of 4. This approach led to the identification of five candidates with homozygous or hemizygous alleles shared by both siblings (Table S1). Subsequent in-depth investigations for those candidates ruled out other loci based on the population frequencies and the genotype-phenotype correlations, suggesting the 2,876 bp deletion located in intron 1 of the *SLC16A2* gene as the most likely cause in these siblings (Fig. 1C). Additionally, we validated its presence in both patients and their parents. A multiplexed PCR method was designed to target the wild-type (WT) allele (F1-R1, band size: 373 bp) and the deleted allele (F1-R2, band size: 852 bp). The genotyping results confirmed the presence of the deleted allele in both patients, while the father carried only the WT allele. Also, the result revealed the carrier status in the mother, displaying both bands from the WT and deleted alleles, compatible with X-linked inheritance (Fig. 1D).

We observed that our case was absent in the gnomAD SV v4.0 database. Nonetheless, we found two deletions in female carriers of African descent that were overlapping with and larger than the deletion found in our case. These two deletions (DEL_CHRX_93EF475A and DEL_CHRX_30C1F462), measured 3.35 and 67.47 kb, respectively, and were located in intron 1 of the *SLC16A2* gene; both were identified as singletons (Figure S4). While two female carriers were observed among 47,973 individuals (allele frequency: 2.0x10^− 5^), no hemizygous deletions overlapping with those identified in our case were detected in a healthy male cohort of 15,073 individuals cataloged in the gnomAD SV database. Since the gnomAD database includes small numbers of Korean individuals, we further investigated whether the deletion is an ancestry-specific CNV by utilizing the dataset from the Korean Genome Project [26]. However, no deletion overlapping with the *SLC16A2* gene was identified in a cohort of 1,094 Korean individuals.

### Regulatory elements located in the deletion

**Fig. 2:**
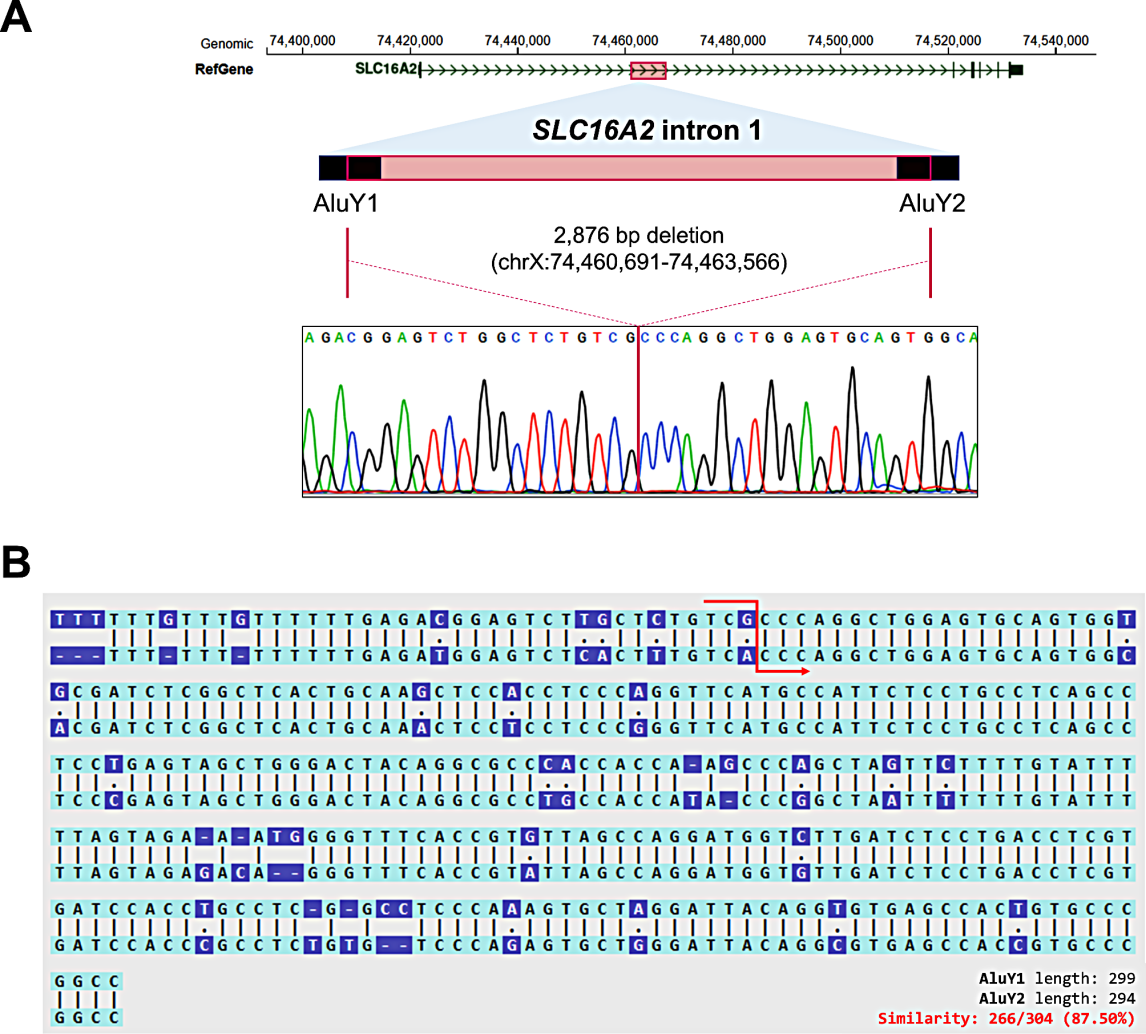
Breakpoint analysis reveals Alu/Alu-mediated rearrangement as the potential generating mechanism of *SLC16A2* intronic deletion. **A**. The deletion is located in the intron 1 of the *SLC16A2* gene and flanked by the AluY1 and the AluY2 repeat sequences. Sanger sequencing confirmed the breakpoints. **B**. High sequence similarity between the two AluY sequences implies Alu/Alu-mediated rearrangement as the responsible generating mechanism of this deletion. Breakpoints are highlighted in red

**Fig. 3:**
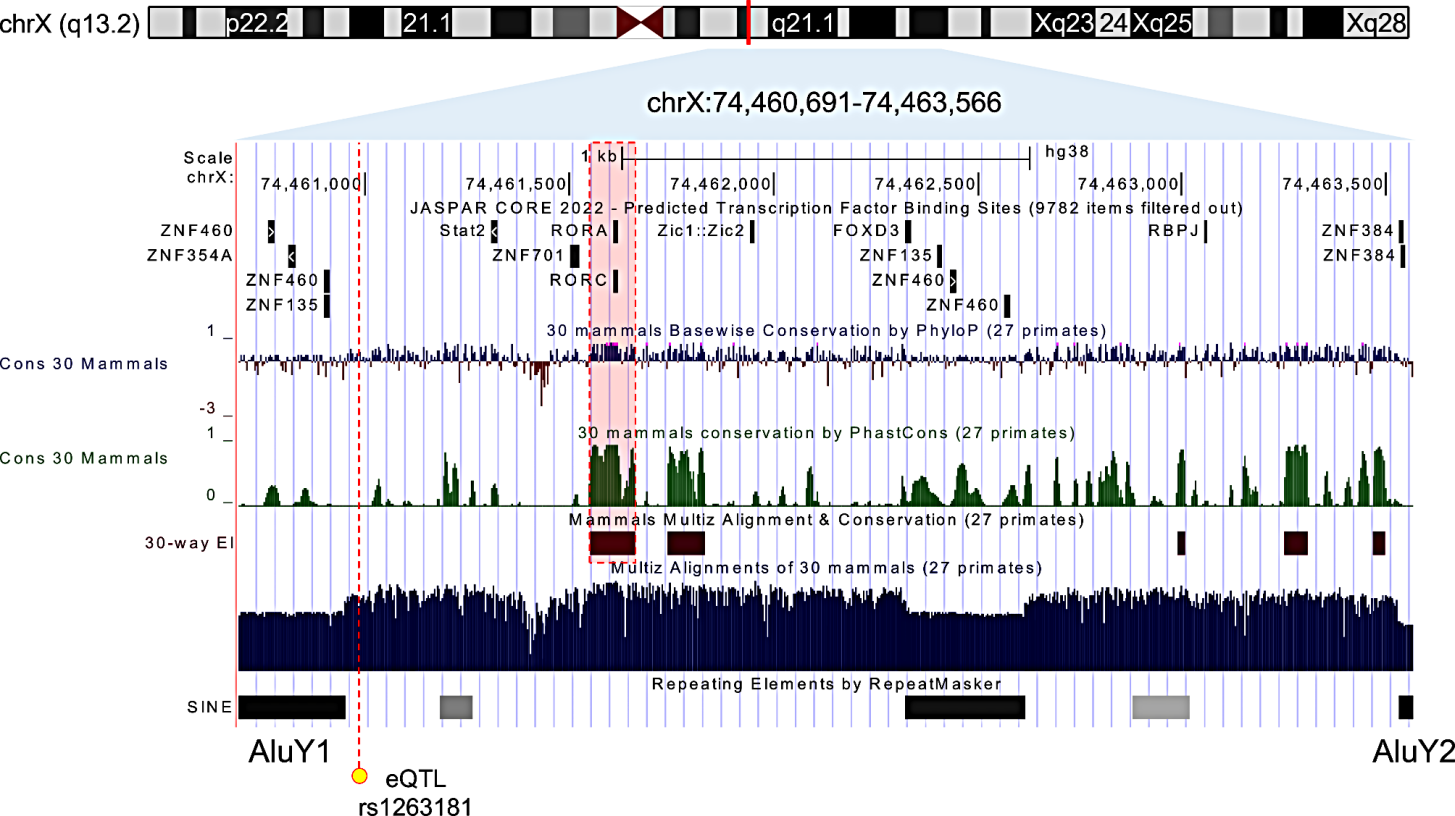
Deleted non-coding region in *SLC16A2* gene exhibiting regulatory features. A detailed genomic overview of a 2.9 kb deletion (chrX:74,460,691 − 74,463,566; hg38) located within intron 1 of the *SLC16A2* gene is presented, flanked by AluY repetitive sequences. The deletion encompasses multiple transcription factor binding sites predicted from the JASPAR database, including those for RORA and RORC, which coincide with regions conserved across 30 mammalian species. An eQTL (rs1263181), identified by GTEx, is indicated by a yellow dot, suggesting a regulatory function. The conservation profile, repetitive elements, and transcription factor sites provide insights into the regulatory potential of the non-coding region

**Fig. 4:**
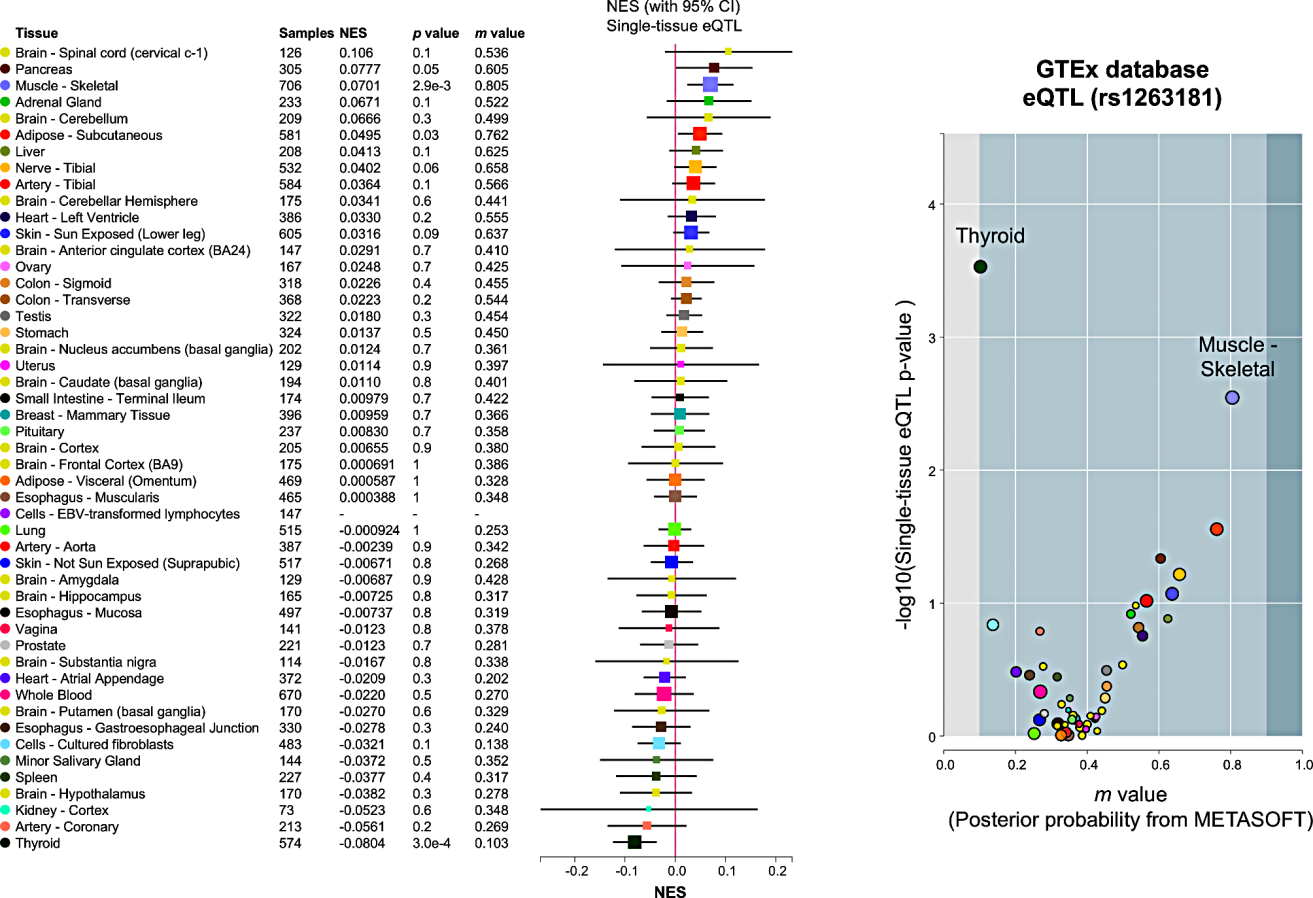
Tissue-specific regulatory impact of eQTL rs1263181 on *SLC16A2* expression levels. The figure displays the differential expression impact of eQTL (rs1263181) across various human tissues, based on GTEx database analysis. The eQTL is significantly associated with gene expression in thyroid and skeletal muscle, implicating its potential involvement in Allan-Herndon-Dudley Syndrome pathophysiology. The left panel enumerates tissues with corresponding normalized effect sizes (NES), p-values, and m-values from METASOFT, indicating the probability of eQTL association. The right panel plots the negative logarithm of the p-value against the m-value for each tissue, highlighting the strongest associations in thyroid and skeletal muscle. Data spans 49 tissues, affirming the eQTL’s variable expression influence

Next, we established the precise breakpoints (chrX:74,460,691 − 74,463,566; hg38) through Sanger sequencing of the PCR product spanning this deletion (Fig. 2A). Notably, the breakpoints were flanked by two AluY sequences, which exhibit a high degree of sequence similarity (Fig. 2B). This suggests Alu-specific microhomology-mediated rearrangement as the potential mechanism generating the deletion between the two AluY sequences [27]. We also assessed the impact of this deletion on aberrant splicing (Figure S2). However, the RT-PCR result indicated that no aberrant splicing occurred between exon 1 and exon 2 attributable to the intronic deletion. Next, we investigated this region using the UCSC genome browser whether it possesses regulatory features (Fig. 3). Notably, the deleted region contains multiple predicted sequences that may serve as binding sites for crucial transcriptional regulators, including Stat2, Zic1, Zic2, FOXD3, and several zinc finger proteins, as identified in the JASPAR database (Table 1) [23]. In particular, the binding sites for RORA and RORC coincide with a region that is highly conserved among 30 mammalian species, suggesting important regulatory functions (Figure S3). Furthermore, we identified an eQTL (rs1263181) in this region, documented in the GTEx database [24]. This eQTL showed the most robust signal with a positive normalized effect size (NES; 0.07) in skeletal muscle tissue (*P* = 2.9x10^− 3^) and a negative NES (-0.08) in thyroid tissue (*P* = 3.0x10^− 4^). However, it did not exhibit significant effects in blood or skin tissues (Fig. 4). Taken together, these findings suggest that the identified non-coding deletion in *SLC16A2* could be a critical region associated with AHDS, likely due to the loss of regulatory elements affecting *SLC16A2* expression.

**Table 1:** Transcription factor binding sites predicted in the deleted region from the JASPAR 2022 database

TF	JASPAR ID	Position (hg38)	Strand	Motif sequence	Score
ZNF460	MA1596.1	chrX:74,460,763–74,460,778	+	gCCTCaGCCTCCCgag	756
ZNF354A	MA1872.1	chrX:74,460,812–74,460,832	-	.taa.tatAAAtGgactaatT	641
ZNF460	MA1596.1	chrX:74,460,898–74,460,913	+	gCCTCaGCCTCCCgag	747
ZNF135	MA1587.1	chrX:74,460,899–74,460,912	+	cCTCGACCTCCtga	630
Stat2	MA1623.1	chrX:74,461,310–74,461,322	-	agAAAcAGAAA.	641
ZNF701	MA1876.1	chrX:74,461,504–74,461,524	+	GAGcAccaaaGGGGGga….	652
RORA	MA0072.1	chrX:74,461,608–74,461,621	-	.atAa.TAGGTCAa	621
RORC	MA1151.1	chrX:74,461,609–74,461,620	-	.tAa.TaGGtCA	632
Zic1::Zic2	MA1628.1	chrX:74,461,942–74,461,952	-	c.CAGCAGG.	662
FOXD3	MA0041.2	chrX:74,462,321–74,462,336	-	c.CAGCAGG.	614
ZNF135	MA1587.1	chrX:74,462,401–74,462,414	+	cCTCGACCTCCtga	632
ZNF460	MA1596.1	chrX:74,462,432–74,462,447	+	gCCTCaGCCTCCCgag	963
ZNF460	MA1596.1	chrX:74,462,565–74,462,580	+	gCCTCaGCCTCCCgag	669
RBPJ	MA1116.1	chrX:74,463,053–74,463,062	+	.TGGGAA.	602
ZNF384	MA1125.1	chrX:74,463,533–74,463,544	-	.taAAAAAAaa	622
ZNF384	MA1125.1	chrX:74,463,535–74,463,546	-	.taAAAAAAaa	611

This table lists the predicted binding sites for various transcription factors within a deleted genomic region, each with a score above 600 (*P*-value ≤ 10^− 6^), according to the JASPAR 2022 database. Each entry includes the genomic position, JASPAR ID, transcription factor (TF) name, strand orientation (+ for sense, - for antisense), the DNA motif sequence, and the prediction score. Lowercase letters in motif sequences indicate a lower confidence in base identification

## Discussion

In this study, we utilized long-read genome sequencing to investigate the genetic cause of two siblings who had remained undiagnosed from exome sequencing. Despite clinical diagnoses, their genetic cause remained elusive for over a decade. The patients exhibited common clinical features such as severe global developmental delay, poor growth, seizures, and post-natal microcephaly, but notable intrafamilial variability was observed. Specifically, only the second child (II:2) presented with the pathognomonic TFT findings of AHDS, while the third child (II:3) showed normal TFT results. Additionally, brain MRI findings were not specific indicators of AHDS, with hypomyelination suspected only in the patient II:3 (Fig. 1B). These findings may be caused by the timing of assessment and the heterogeneous clinical features frequently observed in AHDS [9, 10]. Therefore, it was challenging to pinpoint *SLC16A2* based on clinical findings. However, the pedigree information strongly suggested recessive inheritance (Fig. 1A), allowing us to effectively prioritize SVs with homozygous or hemizygous alleles (Table S1). This familial history offers a valuable strategy to identify genetic causes of recessive conditions in exome-negative cases. While the deletion could have been detected using short-read genome sequencing, long-read HiFi genome sequencing enabled comprehensive genome-wide screening for all types of candidate variants contributing to the disorder, including SVs and STRs. Additionally, it provided precise resolution of the breakpoints, demonstrating its additive value in resolving cases of undiagnosed individuals.

The characterization of the breakpoints of this intronic deletion provides supportive evidence for pathogenic causality. Song et al. demonstrated that pathogenic SVs resulting from Alu/Alu-mediated rearrangements preferentially involve younger Alu elements, such as AluS-AluY and AluY-AluY [27]. Our finding of the deletion occurring between AluY pairs is consistent with this observation, and similar mechanisms have been observed in several diseases, including Fanconi anemia, *SPAST*, and *AAAS* genes [28–30]. Moreover, the region contains multiple predicted TF binding sites, involved in the neurodevelopmental process, such as Stat2, Zic1, Zic2, and FOXD3 (Fig. 3; Table 1). STAT signaling is known to be involved in neuronal and glial differentiation [31], while FoxD3 is involved in the regulation of neural crest formation and migration [32]. In addition, the Zic1 and Zic2 genes are classified as C_2_H_2_-type zinc finger proteins (C_2_H_2_-ZNFs) and are crucial regulators of the hedgehog-signaling pathway and neurogenesis, including neurulation [33]. Recently, C_2_H_2_-ZNFs have been highlighted for their involvement in the pathological processes associated with neurodevelopmental disorders, and ZNF384 is also classified among these C_2_H_2_-ZNFs [34]. Therefore, the loss of this region may disturb the neurodevelopmental process by these critical transcriptional regulators, which provide a delineation for a possible mechanism. Notably, the most highly conserved region in the deleted sequence includes TF binding sites for RORA and RORC (**Figure S4**). The *RORA* gene is known to be associated with intellectual disability with or without epilepsy or cerebellar ataxia, in autosomal dominant inheritance (OMIM #618060) [35]. The clinical features of *RORA*-related disorder overlap with the clinical presentation of our patients, implicating its associated pathways. Hence, the identified region and associated downstream pathways may have a high priority for future research to understand the underlying mechanisms of the intronic deletion in our patients with AHDS.

### Limitation

Limitations of our study include the absence of functional studies establishing a direct causative link between the *SLC16A2* intronic deletion and AHDS. While approaches such as luciferase reporter assays, Chromatin Immunoprecipitation (ChIP)-seq, or animal models could provide functional evidence for the regulatory role of this region, conducting such studies presents significant challenges. These include the need to account for cell-type-specific effects and the complexities of replicating the in vivo context in animal models, which may limit the feasibility of such experiments at this stage. Additionally, while the carrier status of the proband’s asymptomatic mother was verified, the unavailability of male siblings in the mother’s lineage (I:2) limited our capacity to further scrutinize this genetic variant. Specifically, it remained unclear whether the variant was inherited or arose spontaneously as *de novo*. Furthermore, although an X-inactivation assay in the mother could potentially provide functional evidence, conducting this test is currently challenging due to the lack of additional samples. Since skewed inactivation toward the normal allele can evoke AHDS in females [4–6], the asymptomatic status of the mother suggests that observing skewed inactivation toward the deleted allele could provide additional evidence supporting the pathogenicity of the deleted allele.

Additionally, the inability to determine the carrier status of the sister (II:1) restricts the potential for informative genetic counseling in future scenarios. Furthermore, despite our findings associating this genetic region with AHDS, its classification remains as a variant of uncertain significance (VUS) according to the ACMG/ClinGen criteria (evidence categories 1 A, 2B, 3 A, 5D) for CNV interpretation [36]. The ACMG/ClinGen criteria assign high scores to *de novo* variants and those overlapping with previously established regions, yet it did not apply to our case; the application of the ACMG/ClinGen criteria may not be appropriate for identified novel disease loci located in the X chromosome, such as in our case [37]. Due to the challenges for the interpretation of variants in non-coding regions [38], a heuristic scoring system has been developed in the RegulomeDB [25]. According to this system, the identified deletion was categorized into category 1, which is predicted to have the most significant regulatory impact. Specifically, the eQTL (rs1263181), located within the deletion, suggests a tissue-specific regulation of *SLC16A2* expression levels (Fig. 4), with significant associations in skeletal muscle and thyroid tissues but not in whole blood or skin tissues. These effects correlate with the major organs involved in AHDS. These findings are supported by the identification of multiple TF binding sites within the region, highlighting its potential regulatory significance (Table 1). Future research should aim to elucidate the underlying mechanisms by which this intronic deletion is associated with AHDS. Additionally, further case reports involving this region from undiagnosed neurodevelopmental cohorts or patients lacking a genetic diagnosis but presenting with clinical features of AHDS are warranted to delineate the biological role of these regulatory components.

## Conclusion

This study employed long-read HiFi genome sequencing to explore the genetic basis of AHDS in two male siblings with negative exome results. We identified a novel hemizygous 2.8 kb deletion in the non-coding region of *SLC16A2* as a strong candidate for AHDS. Our study highlights the regulatory role of the non-coding region that may underlie the development of AHDS, and our analysis suggests Alu/Alu-mediated rearrangements as a generating mechanism. These findings expand our understanding of the genomic landscape of AHDS and the utility of long-read sequencing for investigating patients with undiagnosed rare diseases. However, this study also highlights the ongoing challenges in interpreting non-coding variants, emphasizing the need for further functional studies.

## Electronic supplementary material

Below is the link to the electronic supplementary material.

Supplementary Material 1

## Data Availability

The datasets generated and/or analysed during the current study are available in the Zenodo repository (https://zenodo.org/records/10957750).
