# Supplementary material for: Identification of a novel non-coding deletion in Allan-Herndon-Dudley syndrome by long-read HiFi genome sequencing
Source: BMC Med Genomics. 2025 Mar 3;18:41. doi: 10.1186/s12920-024-02058-4 (PMC11877835; doi:10.1186/s12920-024-02058-4)
Supplement: Supplementary file 1 — Supplementary Material 1 [file 12920_2024_2058_MOESM1_ESM.pdf]

Supplementary Information

Identification of a novel non-coding deletion in Allan-Herndon-Dudley syndrome by HiFi long-read genome sequencing

Jihoon G. Yoon, Seungbok Lee, Soojin Park, Se Song Jang, Jaeso Cho, Man Jin Kim, Soo Yeon Kim, Woo Joong Kim, Jin Sook Lee, and Jong-Hee Chae

< Table of Contents >

Figure S1. Genotyping analysis of pathogenic short-tandem repeat loci in siblings ….2

Figure S2. Assessment of the *SLC16A2* intronic deletion's effect on mRNA splicing 6

Figure S3. In silico identification of RORA and RORC transcription factor binding sites in conserved genomic regions ……………………………………..……………………….....7

Figure S4. Identification of overlapping deletions in gnomAD SV …………………….....8

Table S1. Catalog of homozygous and hemizygous structural variations in OMIM genes observed in siblings ………...9

Figure S1 | Genotyping analysis of pathogenic short-tandem repeat loci in siblings.

This figure presents the genotyping results from long-read sequencing for 52 known pathogenic short-tandem repeat (STR) sites in two siblings. Results for the second child (II:2, **A**) and the third child (II:3, **B**) show no pathogenic repeat expansions in the known STR disorders. The bars display the number of repeat units (y-axis) for each allele observed at that locus, with the x-axis indicating the specific allele sizes detected. Red and blue bars represent reads with forward and reverse strands, respectively.

A

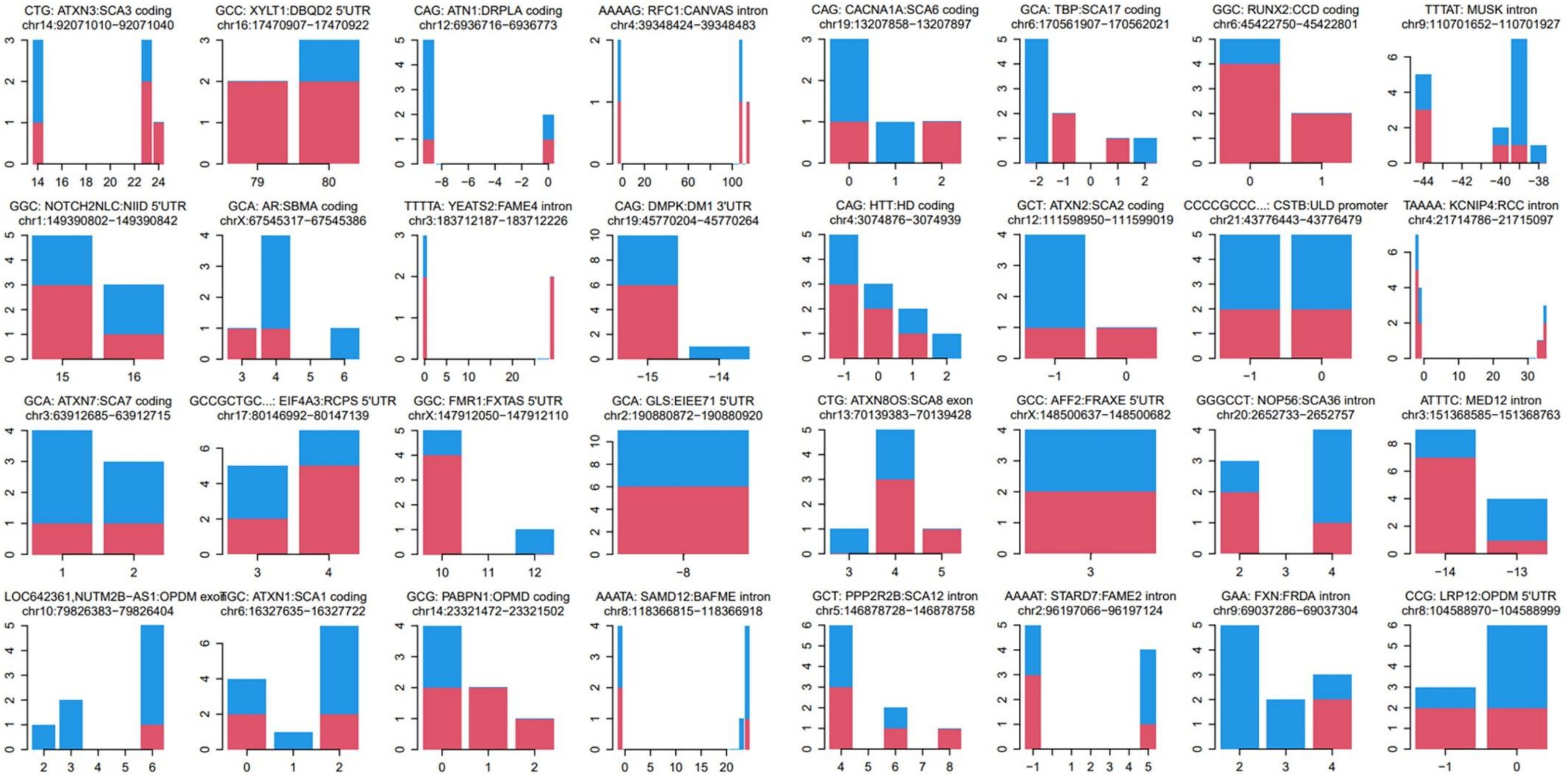

A (continued)

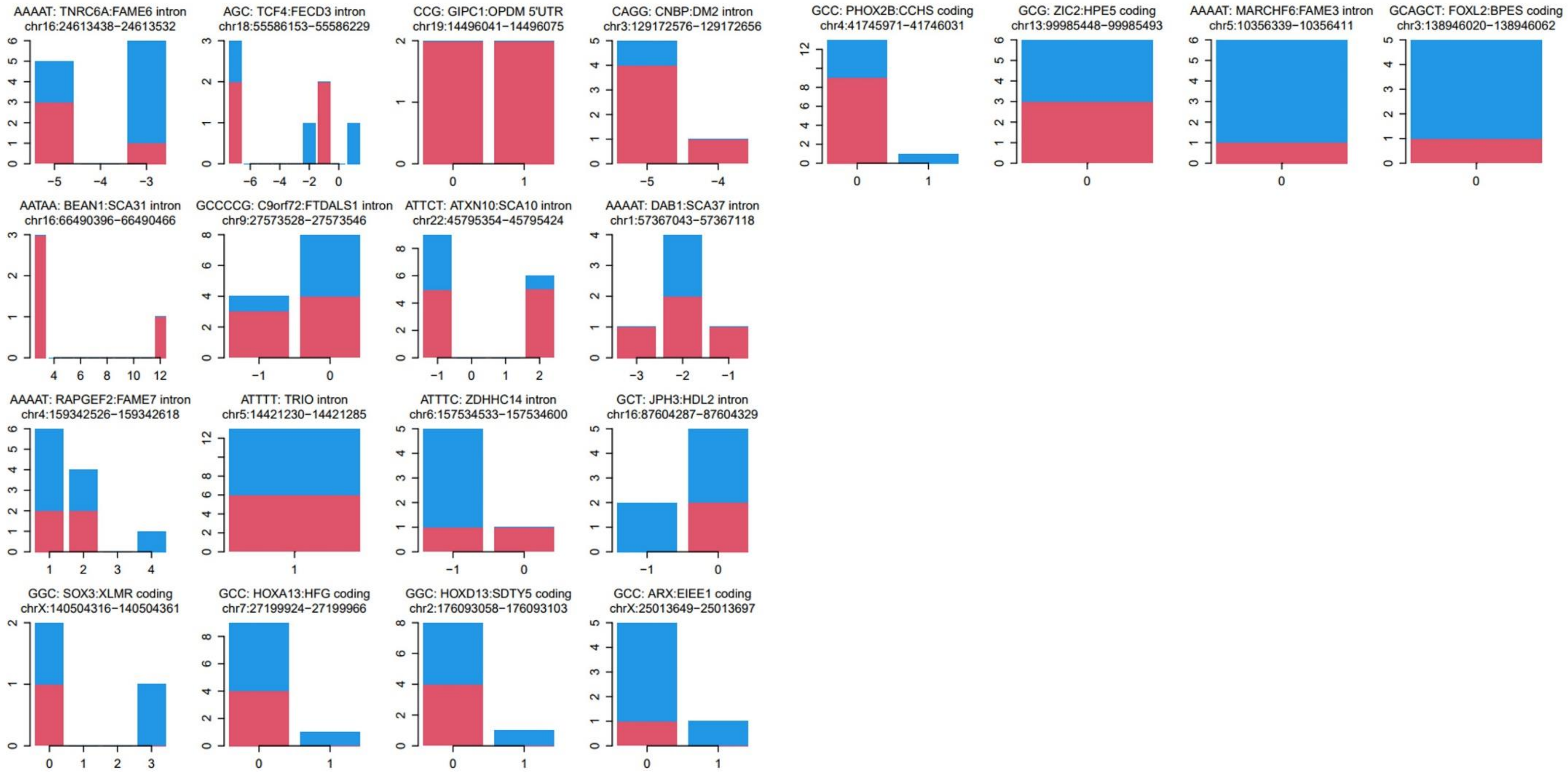

AAAAT: TNRC6A:FAME6 intron
chr16:24613438−24613532
AGC: TCF4:FECD3 intron
chr18:55586153−55586229
CCG: GIPC1:OPDM 5'UTR
chr19:14496041−14496075
CAGG: CNBP:DM2 intron
chr3:129172576−129172656
GCC: PHOX2B:CCHS coding
chr4:41745971−41746031
GCG: ZIC2:HPE5 coding
chr13:99985448−99985493
AAAAT: MARCHF6:FAME3 intron
chr5:10356339−10356411
GCAGCT: FOXL2:BPES coding
chr3:138946020−138946062
AATAA: BEAN1:SCA31 intron
chr16:66490396−66490466
GCCCCG: C9orf72:FTDALS1 intron
chr9:27573528−27573546
ATTCT: ATXN10:SCA10 intron
chr22:45795354−45795424
AAAAT: DAB1:SCA37 intron
chr1:57367043−57367118
AAAAT: RAPGEF2:FAME7 intron
chr4:159342526−159342618
ATTTT: TRIO intron
chr5:14421230−14421285
ATTTC: ZDHHC14 intron
chr6:157534533−157534600
GCT: JPH3:HDL2 intron
chr16:87604287−87604329
GGC: SOX3:XLMR coding
chrX:140504316−140504361
GCC: HOXA13:HFG coding
chr7:27199924−27199966
GGC: HOXD13:SDTY5 coding
chr2:176093058−176093103
GCC: ARX:EIEE1 coding
chrX:25013649−25013697

B

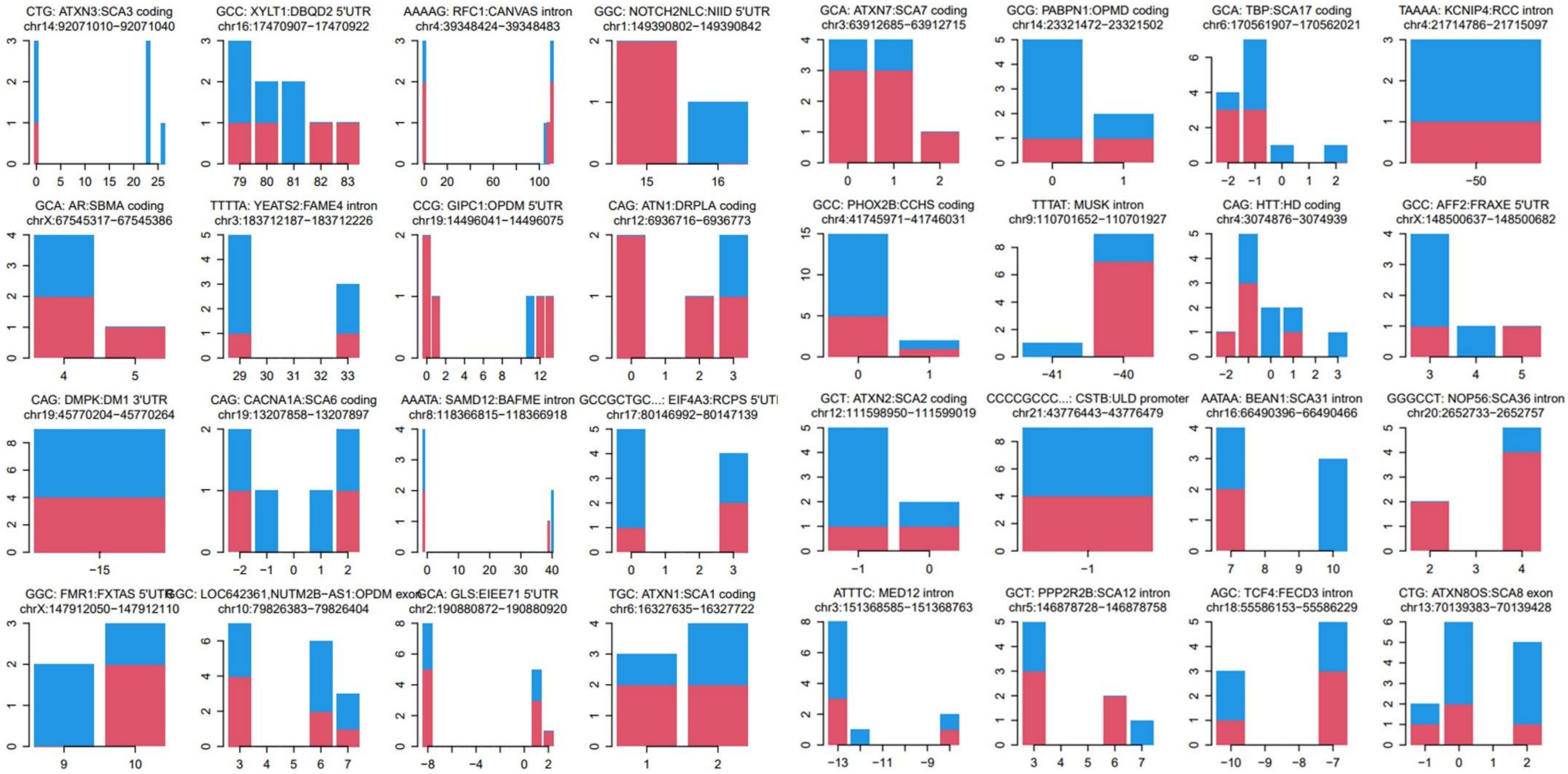
CTG: ATXN3:SCA3 coding
chr14:92071010−92071040
GCC: XYLT1:DBQD2 5'UTR
chr16:17470907−17470922
AAAAG: RFC1:CANVAS intron
chr4:39348424−39348483
GGC: NOTCH2NLC:NIID 5'UTR
chr1:149390802−149390842
GCA: ATXN7:SCA7 coding
chr3:63912685−63912715
GCG: PABPN1:OPMD coding
chr14:23321472−23321502
GCA: TBP:SCA17 coding
chr6:170561907−170562021
TAAAA: KCNIP4:RCC intron
chr4:21714786−21715097
GCA: AR:SBMA coding
chrX:67545317−67545386
TTTTA: YEATS2:FAME4 intron
chr3:183712187−183712226
CCG: GIPC1:OPDM 5'UTR
chr19:14496041−14496075
CAG: ATN1:DRPLA coding
chr12:6936716−6936773
GCC: PHOX2B:CCHS coding
chr4:41745971−41746031
TTTAT: MUSK intron
chr9:110701652−110701927
CAG: HTT:HD coding
chr4:3074876−3074939
GCC: AFF2:FRAXE 5'UTR
chrX:148500637−148500682
CAG: DMPK:DM1 3'UTR
chr19:45770204−45770264
CAG: CACNA1A:SCA6 coding
chr19:13207858−13207897
AAATA: SAMD12:BAFME intron
chr8:118366815−118366918
GCCGCTGC...: EIF4A3:RCPS 5'UTI
chr17:80146992−80147139
GCT: ATXN2:SCA2 coding
chr12:111598950−111599019
CCCCGCCC...: CSTB:ULD promoter
chr21:43776443−43776479
AATAA: BEAN1:SCA31 intron
chr16:66490396−66490466
GGGCCT: NOP56:SCA36 intron
chr20:2652733−2652757
GGC: FMR1:FXTAS 5'UTR
chrX:147912050−147912110
GGC: LOC642361,NUTM2B−AS1:OPDM exon
chr10:79826383−79826404
GCA: GLS:EIEE71 5'UTR
chr2:190880872−190880920
TGC: ATXN1:SCA1 coding
chr6:16327635−16327722
ATTTC: MED12 intron
chr3:151368585−151368763
GCT: PPP2R2B:SCA12 intron
chr5:146878728−146878758
AGC: TCF4:FECD3 intron
chr18:55586153−55586229
CTG: ATXN8OS:SCA8 exon
chr13:70139383−70139428

B (continued)

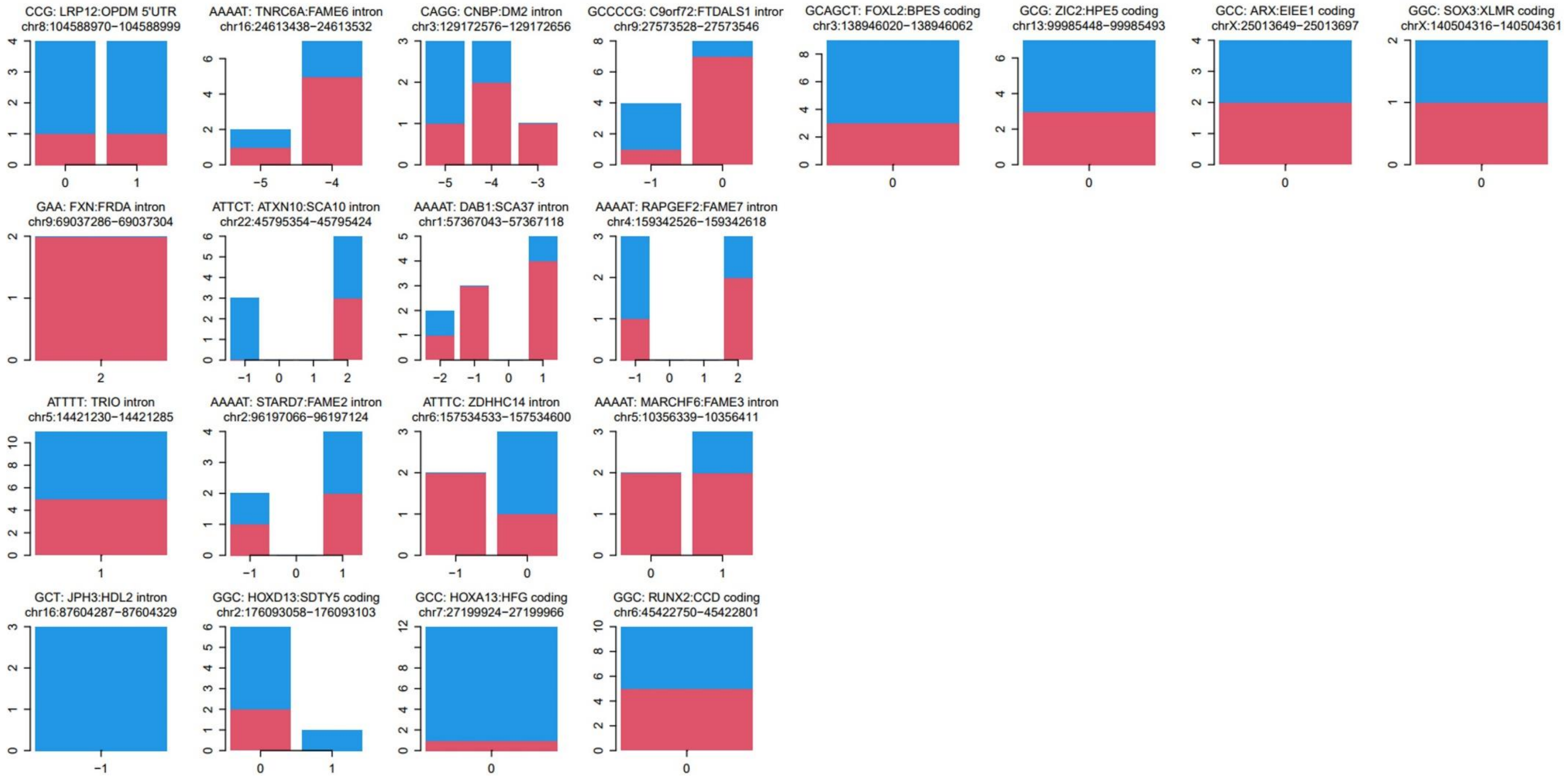
CCG: LRP12:OPDM 5'UTR
chr8:104588970−104588999
AAAAT: TNRC6A:FAME6 intron
chr16:24613438−24613532
CAGG: CNBP:DM2 intron
chr3:129172576−129172656
GCCCCG: C9orf72:FTDALS1 intron
chr9:27573528−27573546
GCAGCT: FOXL2:BPES coding
chr3:138946020−138946062
GCG: ZIC2:HPE5 coding
chr13:99985448−99985493
GCC: ARX:EIEE1 coding
chrX:25013649−25013697
GGC: SOX3:XLMR coding
chrX:140504316−140504361
GAA: FXN:FRDA intron
chr9:69037286−69037304
ATTCT: ATXN10:SCA10 intron
chr22:45795354−45795424
AAAAT: DAB1:SCA37 intron
chr1:57367043−57367118
AAAAT: RAPGEF2:FAME7 intron
chr4:159342526−159342618
ATTTT: TRIO intron
chr5:14421230−14421285
AAAAT: STARD7:FAME2 intron
chr2:96197066−96197124
ATTTC: ZDHHC14 intron
chr6:157534533−157534600
AAAAT: MARCHF6:FAME3 intron
chr5:10356339−10356411
GCT: JPH3:HDL2 intron
chr16:87604287−87604329
GGC: HOXD13:SDTY5 coding
chr2:176093058−176093103
GCC: HOXA13:HFG coding
chr7:27199924−27199966
GGC: RUNX2:CCD coding
chr6:45422750−45422801

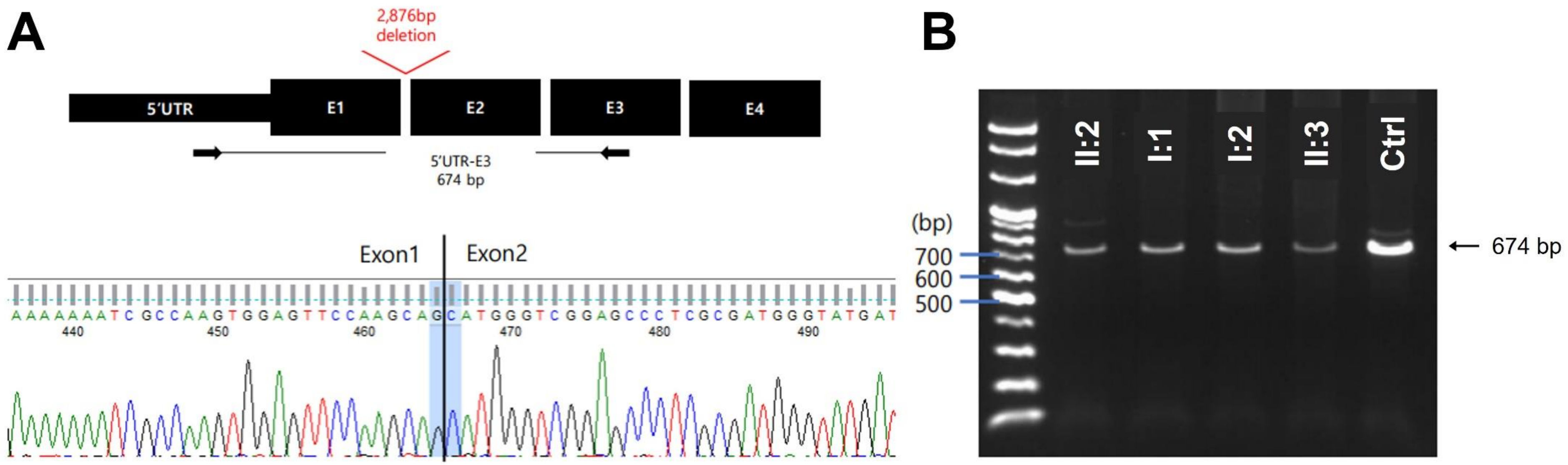

Figure S2 | Assessment of the *SLC16A2* intronic deletion's effect on mRNA splicing.

A. This panel displays a schematic of the *SLC16A2* gene indicating the location of the 2,876 bp intronic deletion and the exons involved. The chromatogram beneath illustrates the boundary between Exon 1 and Exon 2, with no visible signs of splicing anomalies. **B**. This panel depicts an agarose gel electrophoresis of RT-PCR products from blood-derived mRNA, comparing the splicing patterns of siblings II:2 and II:3 with a control. The expected 674 bp band is present in all samples, consistent across both affected siblings and the control, indicating the absence of aberrant splicing events between Exon 1 and Exon 2 attributable to the intronic deletion. This suggests that the intronic deletion does not disrupt the normal splicing of the *SLC16A2* gene in the blood samples tested.

A

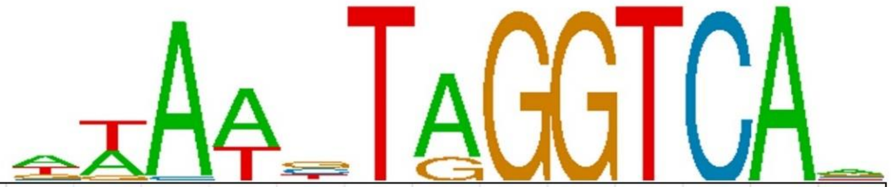

This occurrence	C	A	A	A	A	G	T	A	G	G	T	C	A	C
Motif consensus	.	a	t	A	a	.	T	A	G	G	T	C	A	a
A	0.25	0.47	0.42	0.97	0.64	0.06	0	0.78	0	0	0	0	1	0.42
C	0.22	0.06	0	0.03	0	0.33	0	0	0	0	0	1	0	0.17
G	0.22	0.19	0.08	0	0	0.36	0	0.22	1	1	0	0	0	0.28
T	0.31	0.28	0.5	0	0.36	0.25	1	0	0	0	1	0	0	0.14

B

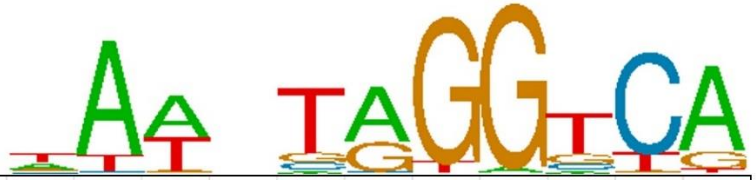

This occurrence	A	A	A	A	G	T	A	G	G	T	C	A
Motif consensus	.	t	A	a	.	T	a	**G**	**G**	t	C	A
A	0.31	0.33	0.86	0.53	0.22	0.04	0.62	0	0.04	0.06	0.01	0.78
C	0.18	0.11	0.01	0.01	0.25	0.09	0.02	0	0	0.11	0.82	0.01
G	0.26	0.14	0.01	0.03	0.28	0.12	0.34	0.92	0.95	0.17	0.02	0.16
T	0.25	0.42	0.12	0.43	0.25	0.75	0.01	0.07	0.01	0.66	0.15	0.04

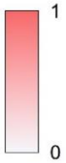

Figure S3 | In silico identification of RORA and RORC transcription factor binding sites in conserved genomic regions.

Sequence motifs derived from the JASPAR database representing predicted binding sites for (A) RORA and (B) RORC within evolutionarily conserved regions of the deleted region. Both motifs are visualized using sequence logos, where the height of each letter correlates with the nucleotide frequency at that position, and the overall height of the stack indicates the sequence conservation. The accompanying tables provide the nucleotide occurrence frequency, with the consensus sequence noted below each table. Red shading highlights nucleotides with a high frequency of occurrence, underscoring their potential importance in transcription factor affinity and specificity. **A**. RORA (RAR-related orphan nuclear receptor alpha), a key regulator implicated in cerebellar development and linked to the Mendelian disorder characterized by intellectual developmental disorder with or without epilepsy or cerebellar ataxia (OMIM #618060). The depicted motif corresponds to the chrX:74461608-74461621 region (JASPAR ID: MA0072.1), with a prediction score of 621, indicating a high likelihood of RORA binding. **B**. RORC (RAR-related orphan nuclear receptor gamma), the motif represents the chrX:74461609-74461620 region (JASPAR ID: MA1151.1), with a prediction score of 632, suggesting a probable RORC binding site.

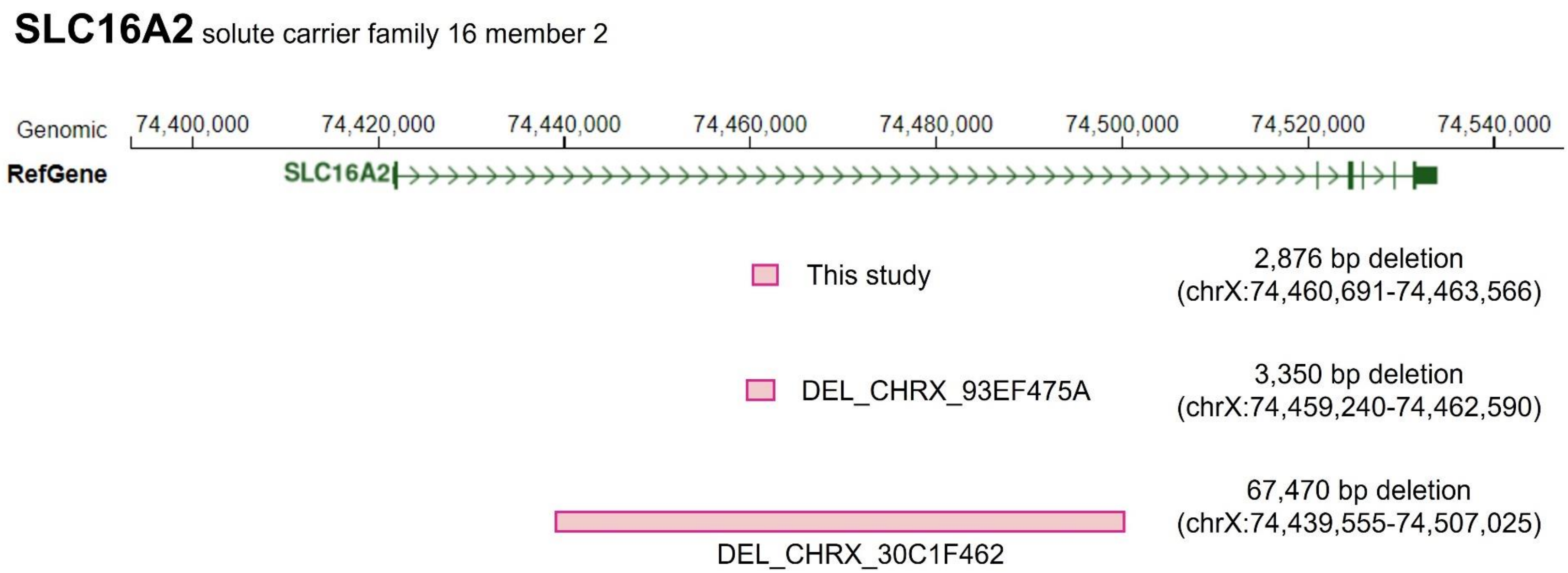

DEL_CHRX_93EF475A

Genetic Ancestry Group	Allele Count	Allele Number	Number of Homozygotes	Number of Hemizygotes	Allele Frequency
African/African American	1	25702	0	0	0.00003891
Amish	0	641	0	0	0.000
Admixed American	0	9102	0	0	0.000
Ashkenazi Jewish	0	2451	0	0	0.000
East Asian	0	3002	0	0	0.000
European (Finnish)	0	4017	0	0	0.000
Middle Eastern	0	46	0	0	0.000
European (non-Finnish)	0	47056	0	0	0.000
Remaining individuals	0	1178	0	0	0.000
South Asian	0	2751	0	0	0.000
XX	1	65800	0	0	0.00001520
XY	0	30146	0	0	0.000
**Total**	**1**	**95946**	**0**	**0**	**0.00001042**

DEL_CHRX_30C1F462

Genetic Ancestry Group	Allele Count	Allele Number	Number of Homozygotes	Number of Hemizygotes	Allele Frequency
African/African American	1	25702	0	0	0.00003891
Amish	0	641	0	0	0.000
Admixed American	0	9102	0	0	0.000
Ashkenazi Jewish	0	2451	0	0	0.000
East Asian	0	3002	0	0	0.000
European (Finnish)	0	4017	0	0	0.000
Middle Eastern	0	46	0	0	0.000
European (non-Finnish)	0	47056	0	0	0.000
Remaining individuals	0	1178	0	0	0.000
South Asian	0	2751	0	0	0.000
XX	1	65800	0	0	0.00001520
XY	0	30146	0	0	0.000
**Total**	**1**	**95946**	**0**	**0**	**0.00001042**

Figure S4 | Identification of overlapping deletions in gnomAD SV.

This figure illustrates two distinct overlapping deletions, along with the intronic deletion found in our study, as cataloged in the Genome Aggregation Database Structural Variant database (gnomAD SV v4.0, available at https://gnomad.broadinstitute.org/). Both deletions were in singletons and identified in female carriers of African descent. The data indicate an absence of hemizygous deletion overlapping with the deletion found in our study within the healthy male population of 15,073 individuals reported in the gnomAD SV database.

Table S1 | Catalog of homozygous and hemizygous structural variations in OMIM genes observed in siblings

Chr	Start	End	Length (bp)	SV type	DP (II:2)	DP (II:3)	Gene	OMIM	MOI
3	190380130	190380131	70	INS	7	11	*CLDN16*	Hypomagnesemia 3, renal (#603959)	AR
16	8711712	8711713	55	INS	4	5	*ABAT*	GABA-transaminase deficiency (#137150)	AR
17	893648	893794	146	DEL	5	5	*NXN*	Robinow syndrome, autosomal recessive 2 (#612895)	AR
X	47148831	47148893	62	DEL	11	4	*RBM10*	TARP syndrome (#300080)	XLR
X	74460690	74463566	2,876	DEL	13	11	*SLC16A2*	Allan-Herndon-Dudley syndrome (#300095)	XLR

Abbreviations: Chr, chromosome; DP, depth; OMIM, Online Mendelian Inheritance in Man; MOI, mode of inheritance; AR, autosomal recessive; XLR, X-linked recessive; INS, insertion; DEL, deletion;
